# The Efficacy of the Combination of Naproxen and Fexofenadine (SJP-003) to Prevent or Reduce Side Effects of Receiving Multiple Travel Vaccines: A Case Report

**DOI:** 10.3390/vaccines10071128

**Published:** 2022-07-15

**Authors:** Pantea Kiani, Jacqueline M. Iversen, Andrew Scholey, Joris C. Verster

**Affiliations:** 1 Division of Pharmacology, Utrecht Institute for Pharmaceutical Sciences (UIPS), Utrecht University, 3584 CG Utrecht, The Netherlands; p.kiani@uu.nl (P.K.); j.c.verster@uu.nl (J.C.V.); 2 Sen-Jam Pharmaceutical, 223 Wall St., #130, Huntington, NY 11743, USA; 3 Nutrition Dietetics and Food, School of Clinical Sciences, Monash University, Melbourne, VIC 3168, Australia; andrew@scholeylab.com; 4 Centre for Human Psychopharmacology, Swinburne University, Melbourne, VIC 3122, Australia

**Keywords:** travel, vaccination, side effects, naproxen, fexofenadine, SJP-003, measles, yellow fever, cholera, willingness to vaccinate

## Abstract

A considerable number of travelers receive multiple travel vaccinations before going on holiday. Here, we present a case report of a 56-year-old male traveler. On day 1, he received vaccinations against influenza, Tdab (tetanus toxoid, reduced diphtheria toxoid, and acellular pertussis), MMR (measles, mumps, and rubella), yellow fever, and cholera. On days 1,3, 5, and 7, he self-administered an oral vaccine against typhoid. Treatment comprised the combination of 220 mg naproxen and 180 mg fexofenadine (SJP-003), to be taken 4h before and 6h after the vaccinations on day 1, and every 12 h thereafter until the end of day 7. Side effects were noted daily, and their severity was scored on a scale ranging from 0 (absent) to 10 (severe). These reports revealed that, except from a slight bruising at the injection site, no side effects were experienced from day 1 to day 4. After the second dose on day 3, treatment was discontinued. Two hours after taking the typhoid vaccine on Day 5, various flu-like symptoms were reported of moderate to high severity, including fever, muscle aches (both with severity score of 8), headache (severity score 7), and nausea (severity score 6). Therefore, at 2 h after typhoid vaccination on day 5, naproxen and fexofenadine were self-administered. At 4 h thereafter, all symptoms were resolved. Treatment was continued at the 12 h schedule. On day 6 and 7, no side effects were reported. Taken together, this case study suggests that the combination of naproxen and fexofenadine was effective in preventing or reducing vaccination side effects. Therefore, more research is warranted to further evaluate the efficacy of SJP-003.

## 1. Introduction

During the coronavirus disease 2019 (COVID-19) pandemic, international traveling was significantly limited. Although some countries closed borders, most other countries imposed a variety of entering requirements related to COVID-19, such as mandatory quarantine, additional insurances, proofs of vaccination and boosters, or negative polymerase chain reaction test (PCR)-tests for SARS-CoV-2 infection [1,2]. In addition to vaccination against SARS-CoV-2, the usual travel vaccinations continue to be required. Currently, international travel is increasing, including visits to tropical destinations [3], back to pre-COVID-19 levels. International travel to destinations that require vaccination comprises considerable number of travelers. For example, in 2019, more than half a million travelers from the United States visited Africa [4]. Travelers health, including acquiring the necessary vaccinations, is therefore an important issue.

Travel vaccines have been used for over 2 centuries [5] and significantly reduce the risk of acquiring a variety of infection diseases [6]. However, whether vaccinations are routine, required, and recommended depends on the travel destination, planned activities, and duration of the stay [7].

Common injected travel vaccinations include MMR (measles, mumps, and rubella), Tdab (tetanus, diphtheria, and pertussis), yellow fever, whereas oral vaccines are administered for typhoid and cholera. Side effects of these travel vaccinations are frequently reported [8]. These can be either local, i.e., at the site of injection, or systemic, i.e., throughout the body. The US Centers for Disease Control and Prevention (CDC) reports that common local side effects of travel vaccination include pain at the injection site, redness, or swelling, whereas common systemic side effects may include mild fever, headache, feeling tired, nausea, vomiting, diarrhea, and stomach pain [8]. However, the presence and severity of side effects may differ between vaccination, and it cannot be predicted beforehand which side effects will be experienced, and to what extent.

The risk of experiencing side effects is the most frequently reported reason of fear against vaccination [9], and the most important reason to refuse vaccinations [10]. The necessity of vaccination may be a reason for some travelers to reconsider their travel plans, whereas others may decide to go on holiday unprotected by not acquiring the vaccinations.

An effective and safe treatment of possible side effects of vaccinations could help to increase the willingness to vaccinate. Although at present such medicines are not available, SJP-003, the combination of a nonsteroid anti-inflammatory drug, NSAID (naproxen) and an antihistamine drug (fexofenadine), is being developed for this purpose. Here, we describe a case study of an individual that used the combination of naproxen and fexofenadine when receiving multiple travel vaccines.

## 2. Methods

This case study comprises a 56-year-old male employee of Sen-Jam Pharmaceutical. He was aware that SJP-003 was under development, and that its constituents, naproxen and fexofenadine, are marketed as over-the-counter (OTC) drugs in the USA. The individual volunteered to use the drug combination, as he had scheduled several travel vaccinations for a planned trip to Africa.

On day 1, he received 2 intramuscular injections. One vaccine was against influenza (Fluzone^®^ quadrivalent by Sanofi Pasteur, Lyon, France), whereas the other vaccine was a booster (Boostrix^®^, GlaxoSmithKline Biologicals, Brentford, UK) against Tdab (tetanus toxoid, reduced diphtheria toxoid, and acellular pertussis). In addition, he received one subcutaneous injection against MMR (measles, mumps, and rubella) and another subcutaneous injection against yellow fever (Stamaril^®^, Sanofi Pasteur). On day 1, he further received an oral cholera vaccine (100cc, Vaxchora^®^, Emergent Travel Health, Inc., Redwood, CA, USA). Finally, he also self-administered an oral vaccine against typhoid (Vivotif^®^, Berna Biotech, Ltd., Bern, Switzerland) on days 1, 3, 5 and 7.

He self-administered a first dose of SJP-003 (220 mg of naproxen sodium and 180 mg of fexofenadine HCL) four hours before vaccinations on day 1. A second dosing was taken 6 h after the vaccinations on day 1, and every 12 h thereafter until the end of day 7. In case no side effects were experienced, treatment could be discontinued. Since the individual self-administered the medication, treatment was not blinded. No ethics approval was needed for the self-administration of these OTC drugs.

The individual described to the corresponding author his experience of side effects temporally associated with vaccination. This was conducted via phone and text messages. The severity of the side effects of the vaccinations were rated on a scale ranging from 0 (absent) to 10 (extreme), and included flu-like symptoms (fever, headache, chills, and muscle aches) and pain at the injection site. The side effects were rated daily, 2 h and 10 h after vaccination.

## 3. Results

**Figure 1:**
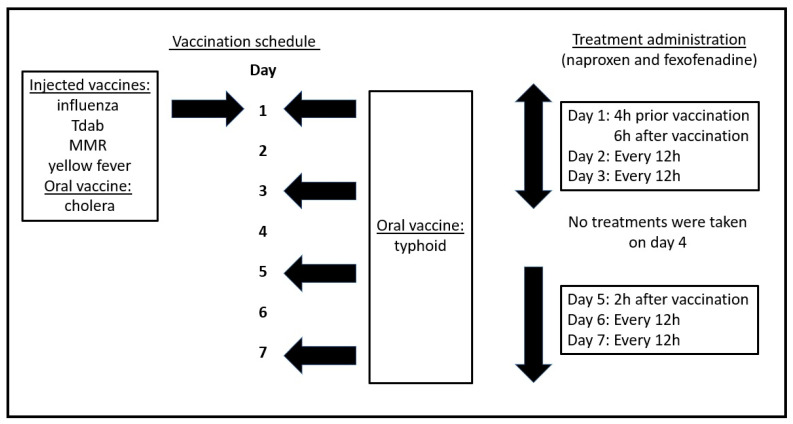
Vaccination schedule and treatment administration. Abbreviations: Tdab = tetanus toxoid, reduced diphtheria toxoid, and acellular pertussis, MMR = measles, mumps, and rubella.

This case was a 56-year-old male individual. He was healthy and had no underlying disease (by self-report). He obtained his travel vaccinations, and an overview of his actual treatment schedule with SJP-003 is given in Figure 1.

He self-administered the first dose of SJP-003 4 h prior to the vaccinations on day 1. He reported a slight bruising at the injection site which lasted until the end of day 2. However, no injection site pain or flu-like symptoms were experienced. A second dose of SJP-003 was taken 6 h after the vaccinations on day 1, and every 12 h thereafter. On day 3, 12 h thereafter, he self-administered his second oral dose of the typhoid vaccine. No flu-like symptoms or other side effects were reported. After day 3, no side effects of the vaccination were experienced, and he discontinued the treatment. On day 5, two hours after self-administering the third oral dose of the typhoid vaccine, he experienced several flu-like symptoms. An overview of all reported symptoms is given in Table 1.

At 2 h after vaccination, he self-administered a single dose of SJP-003. The reported adverse reactions were resolved within 4 h. He continued self-administering SJP-003 every 12 h, through day 7. No side effects were reported on day 6. Additionally, after self-administering the final dose of the oral typhoid vaccine on day 7, no side effects of the vaccine were reported.

## 4. Discussion

The chances of experiencing undesirable side effects from travel vaccinations must be considered as high. For example, the package insert of the oral Vaxchora^®^ cholera vaccine [11] reports on a study among 2789 US and Australian adults, 18 to 45 years old, that the most frequently reported side effects over a 7-day period were tiredness (31.3%), headache (28.9%), abdominal pain (18.7%), nausea and/or vomiting (18.3%), and lack of appetite (16.5%). For the Boostrix^®^ Tdab subcutaneously injected vaccination, the package insert reports on a study among 1480 19-to-65-year-old individuals [12] which also reported high percentages of side effects, including pain (61%), redness (21%), and swelling (18%) at the site of injection, and systemic effects such as headache (30%), fatigue (28%), gastrointestinal symptoms (16%), and fever (6%). These high percentages of side effects do not contribute to the willingness among the general public to get vaccinated. The recommended solution by the Immunization Action Coalition (IAC) to manage and reduce these side effects, i.e., to apply a cold compress to the injection site or consider an OTC pain reliever [13]. It is unlikely that this advice is sufficiently reassuring to convince people that hesitate about getting vaccinated to do so. Instead, it is believed that the availability of an effective and safe medicine that prevents side effects from occurring will be of significantly more use to increase willingness to vaccinate.

This case report illustrates the fact that usually multiple travel vaccines are administered in combination This common practice increases the likelihood of experiencing undesirable side effects, and probably also their severity and duration. This case report suggests that SJP-003 was effective in preventing and reducing side effects of travel vaccinations. The side effects of vaccination on Day 5, although of considerable severity, were resolved within 4 h after taking SJP-003.

Naproxen is a commonly used OTC drug for pain relief and its anti-inflammatory properties are well documented [14]. Fexofenadine is an H1-antagonist with antihistaminergic properties [15]. As such, it was expected that fexofenadine would reduce vaccination side effects such as rash and itch. In addition, anti-inflammatory properties have also been demonstrated for fexofenadine [16]. Given the different mechanisms and different activity profiles of the two drugs, the administration of both naproxen and fexofenadine (SJP-003) may have an additive or synergistic effect in preventing or reducing common vaccination side effects such as pain and flu-like symptoms.

However, it must be stressed that this is only a single case study, and all data were self-reported. Therefore, the efficacy of SJP-003 should be confirmed by double-blind, randomized, placebo-controlled clinical trials. These studies should also confirm that SJP-003 has no clinically relevant impact of vaccine efficacy.

## Figures and Tables

**Table 1:** Side effects reported after oral typhoid vaccination on day 5.

Side Effect on Day 5	Severity Scores	
Side Effect	Within 2 h After Vaccination (No Treatment)	6 h After Vaccination (4 h After SJP-003)
Fever	8	0
Chills	8	0
Muscle aches	8	0
Headache	7	0
Nausea	6	0
Muscle weakness	4	0

SJP-003 was taken 2 h after oral typhoid vaccination. Severity was rated on a scale ranging from 0 (absent) to 10 (extreme).

## Data Availability

All available data are presented in this publication.
